# Performance of molecular and serologic tests for the diagnosis of scrub typhus

**DOI:** 10.1371/journal.pntd.0008747

**Published:** 2020-11-12

**Authors:** Kavitha Kannan, Rebecca John, Debasree Kundu, Divya Dayanand, Kundavaram P. P. Abhilash, Alice Joan Mathuram, Anand Zachariah, Sowmya Sathyendra, Samuel G. Hansdak, O. C. Abraham, Karthik Gunasekaran, Ramya Iyadurai, Asha M. Abraham, John Antony Jude Prakash, Binesh Lal Yesudhason, Balaji Veeraraghavan, M. L. Kavitha, Linda R. Jose, M. N. Sumana, Kavitha Saravu, George M. Varghese

**Affiliations:** 1 Department of Infectious Diseases, Christian Medical College, Vellore, Tamil Nadu, India; 2 Department of Emergency Medicine, Christian Medical College, Vellore, Tamil Nadu, India; 3 Department of Medicine, Christian Medical College, Vellore, Tamil Nadu, India; 4 Department of Clinical Virology, Christian Medical College, Vellore, Tamil Nadu, India; 5 Department of Microbiology, Christian Medical College, Vellore, Tamil Nadu, India; 6 Department of Haematology, Christian Medical College, Vellore, Tamil Nadu, India; 7 JSS Medical College, Mysuru, Karnataka India; 8 Department of Infectious Diseases, Kasturba Medical College, Manipal, Manipal Academy of Higher Education, Karnataka, India; University of Oxford, UNITED KINGDOM

## Abstract

Diagnosis of scrub typhus, caused by the bacterium *Orientia tsutsugamushi*, is challenging because of the overlap of its non-specific symptoms with other infections coupled with the lack of sufficient data on the performance of diagnostic tests. Early diagnosis of scrub typhus is crucial to improve outcomes and this study evaluates the diagnostic performance of various tests. The present study aims at assessing the accuracy of various rapid diagnostic tests, serologic tests, and nucleic acid amplification methods on well-characterized patient samples. Adult patients with acute febrile illness and manifestations suggestive of scrub typhus confirmed by positive PCR in the blood, eschar or tissue were characterized as cases. Patients with acute febrile illness and a confirmed alternate etiology such as culture-confirmed typhoid, smear/PCR positive for malaria, PCR/NS1 antigen positive for dengue, PCR positive for influenza, PCR/MAT positive for leptospirosis, PCR positive for spotted fever were characterized as controls with other infections. The healthy controls consisted of subjects from the same geographic region. We performed the following tests on blood samples for scrub typhus and calculated the sensitivity, specificity, positive predictive value, and negative predictive value: (1) Quantitative real time PCR using 47kDa gene (qPCR); (2) Conventional PCR using 56kDa gene (cPCR); (3) Loop-mediated isothermal amplification assay (LAMP assay); (4) Immunofluorescence assay (IFA); (5) Enzyme-linked immunosorbent assay (ELISA); (6) Weil-Felix test(WF test); and (7) Immunochromatographic Rapid Diagnostic Test (RDT).Among the 316 participants, 158 had confirmed scrub typhus (cases) and 158 were controls. ELISA and RDT detecting *Orientia tsutsugamushi* specific IgM antibodies had excellent discriminative potential with sensitivities and specificities of 92%, 94% and 92%, 92% respectively. The sensitivity and specificity of IFA were found to be 95% and 74% respectively. IgM serology had a false positivity rate of 8% with other acute febrile illnesses such as dengue, leptospirosis and spotted fever due to the nonspecific binding of the pentavalent IgM. LAMP assay had 91.7% sensitivity and 77.2% specificity while qPCR provided excellent sensitivity (97%) and perfect specificity. In conclusion, ELISA and RDT detecting *Orientia tsutsugamushi* specific IgM antibodies have excellent sensitivity and specificity while the accuracy of IFA is suboptimal for the diagnosis of scrub typhus. Given its perfect specificity and superior sensitivity, qPCR is preferred for diagnostic confirmation in reference laboratories particularly for diagnosis of early disease with less than 7 days duration. This study provides a comprehensive evaluation of all currently available diagnostic tests for scrub typhus.

## Author summary

Scrub typhus, a life-threatening vector borne rickettsial infection accounts for a million cases annually. While the disease was initially described predominantly in Asia, Northern Australia and islands in the Indian and Pacific Oceans (known as the tsutsugamushi triangle), scrub typhus has now been reported in areas considered previously free of disease like Africa and South America. International travel also accounts for the microbial trafficking of this disease and its presentation to non-endemic areas. The diagnosis of scrub typhus is challenging due to its non-specific symptoms and the lack of sufficient data on the performance of various diagnostic tests. Several tests have been developed for the diagnosis of scrub typhus over the last few decades. However, there exists ambiguity on which tests are most suitable in a given clinical scenario and the apt timing to perform these tests. This study provides further insight into the performance of various serological and molecular tests in the diagnosis of scrub typhus, assists in understanding their discriminatory potential and diagnostic accuracies enabling prompt treatment of the disease.

## Introduction

Scrub typhus, caused by the bacterium *Orientia tsutsugamushi* and transmitted by the bite of the larval stage of trombiculid mites (chiggers), is the most common and clinically important rickettsial infection worldwide, especially in several Asian countries including India. An estimated one billion people are at risk in endemic regions, with nearly a million cases occurring each year [1]. As most cases occur in rural areas with poor diagnostics, this is almost certainly a gross under estimation. Sero-epidemiologic studies estimate an increasing prevalence ranging from 9–31% across Asia [2, 3]. The symptoms and signs of scrub typhus are non-specific and often resemble other tropical infections such as dengue, malaria, typhoid and leptospirosis which are endemic to these regions. Scrub typhus typically presents with an acute undifferentiated febrile illness which may be associated with headache, cough, shortness of breath, and altered sensorium. Presence of a pathognomonic eschar ranges from 10 to 90% [4, 5]. Acute complications include jaundice, pneumonitis, acute respiratory distress syndrome, septic shock, myocarditis, and meningoencephalitis with one third of patients developing multi-organ dysfunction [6]. Untreated, the case fatality rate can be as high as 30–50% [7].

The diagnosis of scrub typhus is hampered due to its nonspecific clinical presentation, poor awareness and insufficient evidence on the diagnostic accuracy of available tests. Serological assays are easy to perform and are considered the mainstay of diagnosis. Immunofluorescence assay (IFA) for the detection of *O*. *tsutsugamushi* antibodies is considered the standard serological test [8]. However, a major drawback of this technique is the requirement for fluorescent microscopes and expertise in performance and interpretation of the test which are usually not available in endemic areas. The enzyme linked immunosorbent assay (ELISA) to detect *O*. *tsutsugamushi-specific* IgM using recombinant antigen, r56 was reported to have a sensitivity and specificity of 96·3% and 99%, respectively, when compared with IFA [9]. However, ELISA is limited by the requirement of a good laboratory, its cost and is also not feasible as a point-of-care test, especially in rural areas. The rapid diagnostic test which can be used as point of care test in primary health centers showed varying sensitivity and specificity [10–12]. The Weil–Felix agglutination test, the older test, while being a cheap option for diagnosis of rickettsial infections in resource-poor settings has poor sensitivity and specificity and is hence not preferred [13]. The IgM antibody formation after the onset of illness and resultant positivity of various serological tests takes about 5 or 6 days. An additional challenge in the performance of the serological tests is that multiple antigenic variants in *O*. *tsutsugamushi* exist, largely caused by changes in the outer membrane protein, 56-kDa type-specific antigen [14]. Molecular tests will fill this gap by detecting the pathogen early in the disease. PCR assays, conventional or real-time, targeting various genes have been tried and reported to have specificity approaching 100% while sensitivity of PCR varied between conventional, nested and quantitative real time PCR (qPCR) [15, 16]. Loop-mediated isothermal amplification (LAMP) has the advantage that it can be performed using simpler equipment and the entire process takes place at the same temperature unlike PCR, but has a poor sensitivity that varies between 40–56% [13, 17].

There is a definite dearth of information on how well the serological and molecular tests perform in routine clinical settings for the diagnosis of scrub typhus. A better understanding on the discriminatory potential of the available diagnostic tests will assist in choosing the most appropriate investigation for a given clinical setting. This study is performed on well characterized cases of scrub typhus and controls with alternate confirmed febrile illness such as dengue, malaria, typhoid, influenza and leptospirosis with the aim to identify the diagnostic performance of various serological and molecular tests available in making a diagnosis of scrub typhus.

## Methodology

### Patient recruitment and sample collection

Adult patients above 18 years of age with acute undifferentiated fever of 3–21 days duration between July 2017 and March 2019 were evaluated prospectively. A thorough history and examination were carried out and the patient’s signs and symptoms were documented using a predesigned proforma. These patients were investigated and managed as is the routine practice by the attending physician for common febrile illnesses like dengue, scrub typhus, malaria, typhoid, etc. Patients with malignancy, immunosuppression, and autoimmune diseases were excluded from the study. A written informed consent was obtained prior to their inclusion into the study. Additional 5 ml of blood was collected in sterile EDTA tubes, and the scab of the eschar was collected in absolute alcohol, and transported to the laboratory for further testing. The blood sample was centrifuged to separate plasma and buffy coat and was stored at -80°C until further testing. The study was approved by the institutional review board and ethics committee (No. 8626/27.01.2017).

### Cases and controls

Patients with scrub typhus confirmed using 56kDa type specific antigen (TSA) conventional PCR (cPCR) and/or 47kDa htrA (high temperature requirement A) qPCR on blood buffy coat, eschar or any tissue samples were chosen as cases. Patients with acute undifferentiated febrile illness and a confirmed alternate etiology such as culture-confirmed typhoid, smear/PCR positive for malaria, PCR/NS1 antigen positive for dengue, PCR positive for influenza, PCR/MAT positive for leptospirosis, PCR positive for spotted fever were characterized as controls with other infections. The healthy controls consisted of subjects from the same geographic region.

### DNA amplification by conventional PCR and quantitative real time PCR

DNA was extracted from the blood buffy coat, homogenized eschar or tissue samples by using the QIAamp DNA Mini Kit (QIAGEN GmbH, Hilden, Germany) according to the manufacturer’s instructions. A standardized conventional PCR (cPCR), targeting the 56kDa protein gene, was performed using the primers OtsuF and OtsuR (Sigma Aldrich, Bangalore, India) (Table 1) and DNA templates from all samples as described earlier [18]. The 410 bp amplicon was visualized by electrophoresis.

**Table 1:** Primers used for the Scrub typhus.

Name	Length (bp)	Tm	Sequence 5'—3'
**Primers used for conventional PCR (cPCR)**			
**OtsuF**	**20**	**58**	5′-AATTGCTAGTGCAATGTCTG-3
**OtsuR**	**19**	**56**	5′-GGCATTATAGTAGGCTGAG-3′
**Primers used for quantitative real time PCR (qPCR)**			
**Otsu47KDa FP**	**28**		5’- AACTGATTTTATTCAAACTAATGCTGCT-3’
**Otsu47KDa FP**	**30**		5’-TATGCCTGAGTAAGATACRTGAATRGAATT-3’
**Otsu47KDa Probe**	**31**		5’-FAM-TGGGTAGCTTTGGTGGACCGATGTTTAATCT-TAMRA-3’.
**Primers used for loop-mediated isothermal amplification assay (LAMP)**			
**F3**	21	55.1	TGACCGYGGATATATATCACA
**B3**	20	55.5	CAATGCRGTAAGAGCTTCTC
**FIP(F1c-F2)**	23–22	56.3–60.1	GCACTGTAGATACCTTCTGATCCAATACTTTGCAACRAATCGTGAA
**BIP(B1c-B2)**	20–20	55.6–61.8	CCACTKGTTCCTGTGCTTGACGTCTACATCATCAGCAATCA
**LF**	26	58.5	GGATTTTCAAATTCRGTAATCATCTT
**LB**	20	59.8	CTCAYACTGGCAAGCCATTA

Quantitative real-time PCR (qPCR) targeting the 47kDa protein gene was done using primers Otsu 47 kDa FP and Otsu 47kDa RP and Otsu 47kDa probe (Table 1) as detailed by Jiang *et al* [19]. Standard and negative controls were included in each run.

### LAMP assay

Loop mediated isothermal amplification (LAMP) assay for scrub typhus was performed using primers F3 and B3 (5 pmol), FIP and BIP (40 pmol), Loop-F and Loop-B (20 pmol) (Sigma-Aldrich) (Table 1) targeting the *groEL* gene. The results were interpreted by observing the turbidity caused by precipitation of magnesium pyrophosphate [17, 20].

### ST IgM InBios ELISA

ST Detect IgM ELISA kit (InBios International, Seattle, USA) was used to detect anti-56KDa protein antibody. Serum samples were first diluted 1:100 with the sample diluent and added to the micro-wells. The plates were washed and incubated with conjugate. Finally, the substrate solution was added and the reaction was stopped by adding the stop solution. The absorbance was read using an ELISA plate reader at a wavelength of 450 nm. Samples with Optical Density (OD) values of ≥0·8 were taken as positive for Scrub typhus [21].

### ST IgM Indirect Immunofluorescent antibody assay (IFA)

ST IFA slides (OTM-120 Fuller Laboratories, Fullerton, California, USA) coated with four different prototype antigens of *O*. *tsutsugamushi*, namely Karp, Kato, Gilliam, and Boryong were used. Patients’ sera (diluted 1:64 in IgM serum diluent) was added to the IFA slides and further the conjugate was added to label the antigen-antibody complexes. The slides were subsequently washed, dried and observed microscopically (100x magnification) (Carl Zeiss MicroImaging GmbH, Göttingen, Germany). As per the kit, the cut-off titer of ≥1:64 for IFA IgM was considered positive. Positivity in any one or more of the four serotypes was taken as IFA positive.

### Weil Felix Test

Serological testing of OXK, OX19 and OX2 antibodies were carried out as per standard protocol. The titre value of ≥1:80 for OXK was taken as positive for scrub typhus.

### Rapid Diagnostic Tests (RDT)

1. **InBios rapid test for scrub typhus**
  It is an immunochromatographic strip test designed for the qualitative detection of IgM specific to *O*. *tsutsugamushi*. The kit consists of ready-to-use antigen-coated strips and reagents. The color intensity developed by the reaction on the strip is directly related to the concentration of the antibody.
2. **Standard Diagnostics (SD) BioLine Tsutsugamushi-Assay**
  It is a solid phase immuno-chromatographic assay (SD BioLine, Korea) for the rapid, qualitative detection of IgG/IgM/IgA antibodies specific to *O*. *tsutsugamushi* in human serum, plasma, and whole blood.
3. **ImmuneMed Scrub Typhus Rapid**
  It is a lateral flow, immunochromatographic test designed for the qualitative detection of IgM as well as IgG specific to *O*. *tsutsugamushi*. It consists of a mixture of cr56, r21, and kr56 antigens of *O*. *tsutsugamushi*.

The readings for all the laboratory tests with observer variations were carried out by three independent observers blinded to each other’s readings. To avoid any potential bias in the interpretation of results, the clinical status of the patient, his/her reference standard results, and the results of other tests were masked from the assessors.

### Statistical analysis

The diagnostic tests for scrub typhus (PCR, LAMP, ELISA, IFA, Weil Felix Test, RDT) were assessed for sensitivities, specificities, positive predictive value (PPV), negative predictive value (NPV), likelihood ratios and odds ratio [with 95% confidence intervals (CI)]. Significance was assigned at p<0·05 for all parameters and were two-sided unless otherwise indicated. All statistical analysis was performed using SPSS (IBM SPSS Statistics version 14.1).

## Results

Among 316 individuals included in the study, 158 were cases, 118 were controls having acute undifferentiated fever with a confirmed etiology and 40 were healthy controls. There were 79 males (50%) among the cases and 98 male (62%) in the control group. The mean age of patients was 46 years among the cases and 40 years among the controls. The patient characteristics are detailed in Table 2.

**Table 2:** Patient characteristics.

Patient Characteristics	Cases (N = 158)	Controls (N = 158)	p value
Age, Years, Mean ± SD	46·1 ± 15·0	40·3 ± 19·5	0.005
Sex, Male	79 (50)	98 (62)	0.041
Occupation: Agriculture / daily labour	65 (41)	44 (28)	0.342
Duration of illness before admission (days)	9 (3–20)	6 (3–18)	0.142
Fever present at admission (%)	157 (99·4)	98/118 (83%)	<0.001
Eschar present (%)	125 (79·1)	0 (0·0)	<0.001
WBC Count, mean ± SD	10175±4885	7314±5569	<0.001
Platelet Count, mean ± SD	78562±65987	121608±125119	0.001
Total Bilirubin, mg/dl, mean ± SD	2·10±2·59	1·76±2·21	0.243
Direct Bilirubin, mg/dl, mean ± SD	1·51±2·32	0·87±1·52	0.04
Total Protein, g/dl, mean ± SD	6·44±0·97	6·57±0·81	0.483
Albumin, g/dl, mean ± SD	3±2·45	3·58±0·63	<0.001
AST, IU/ l, mean ± SD	170·48±318·24	159·14±276·85	<0.001
ALT, IU/ l, mean ± SD	101·12±129·41	118·50±334·15	<0.001
Alkaline Phosphatase, mean ± SD	189·92±137·73	138·36±143·85	<0.001
Serum Creatinine, mg/dl, mean ± SD	2·2±6·61	1·04±0·51	0.251

SD, standard deviation; N, sample size; AST, aspartate transaminase; ALT, alanine transaminase

**Fig 1:**
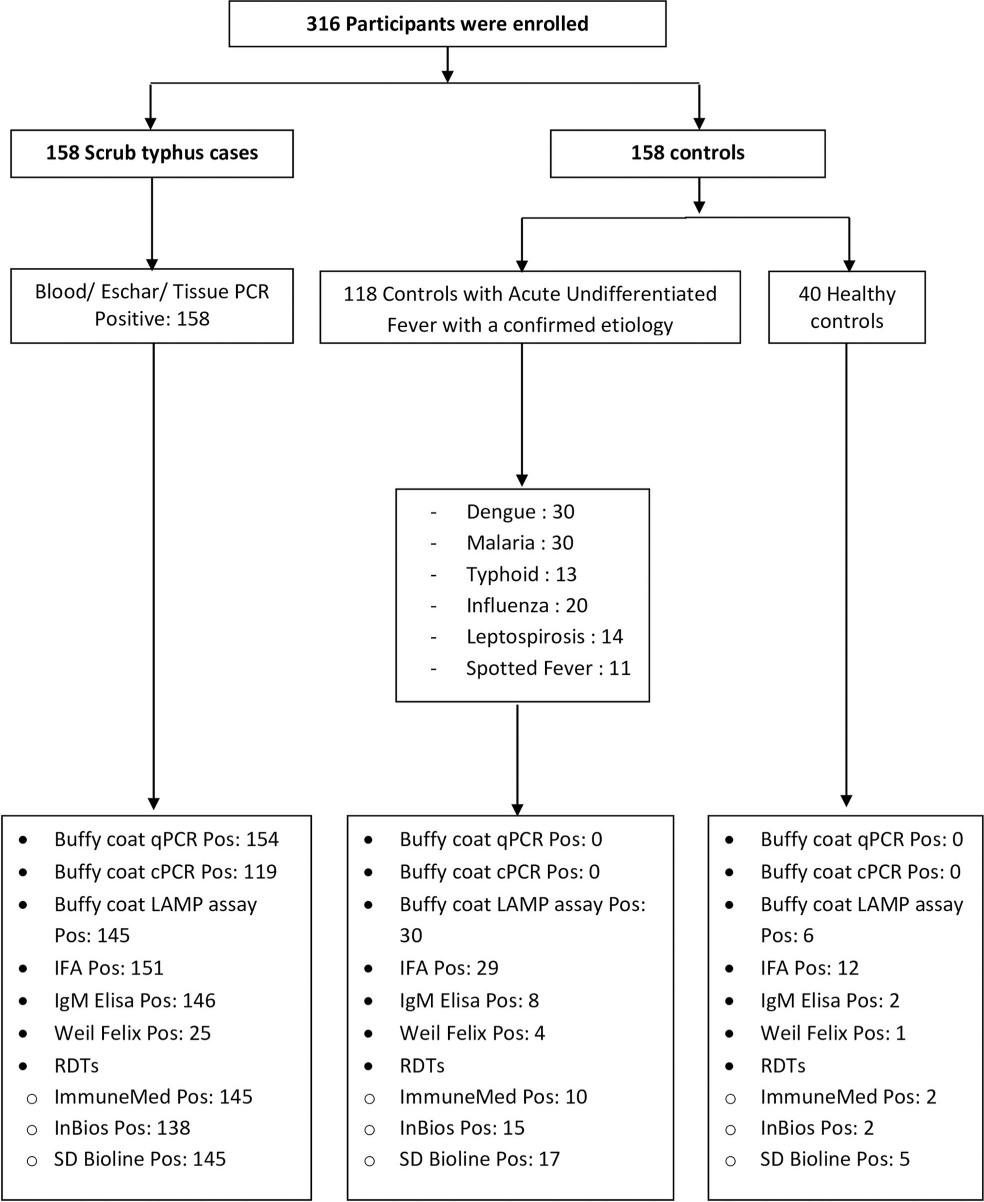
Schematic representation of the study. This figure details the patient enrolment, diagnostic test performed and positive results.

Among the controls with acute undifferentiated fever, patients had the following confirmed etiologies: dengue (30), malaria (30), typhoid (13), influenza (20), leptospirosis (14) and spotted fever (11). The details are presented in Fig 1.

A higher level of total leucocyte count (TLC), 10175±4885 was noted among the cases as opposed to the controls 7314±5569. Similarly lower platelet counts were noted among patients with scrub typhus (78562±65987 vs 121608±125119). The average total bilirubin was 2·10±2·59 among cases as compared to 1·76±2·21 in the control group. Marginal difference in the mean ALT and AST levels was noted between the cases and controls. (AST, 170.48±318.24 among cases and 159·14±276·85 in the control group; ALT, 101·12±129·41 among the cases and 118·50±334·15 among controls). Alkaline Phosphatase was notably higher in the group of patients with scrub typhus (189·92±137·73 vs. 138·36±143·85). Serum creatinine level was also elevated among the cases (2·02±6·61) compared to controls (1·04±0·51).

**Fig 2:**
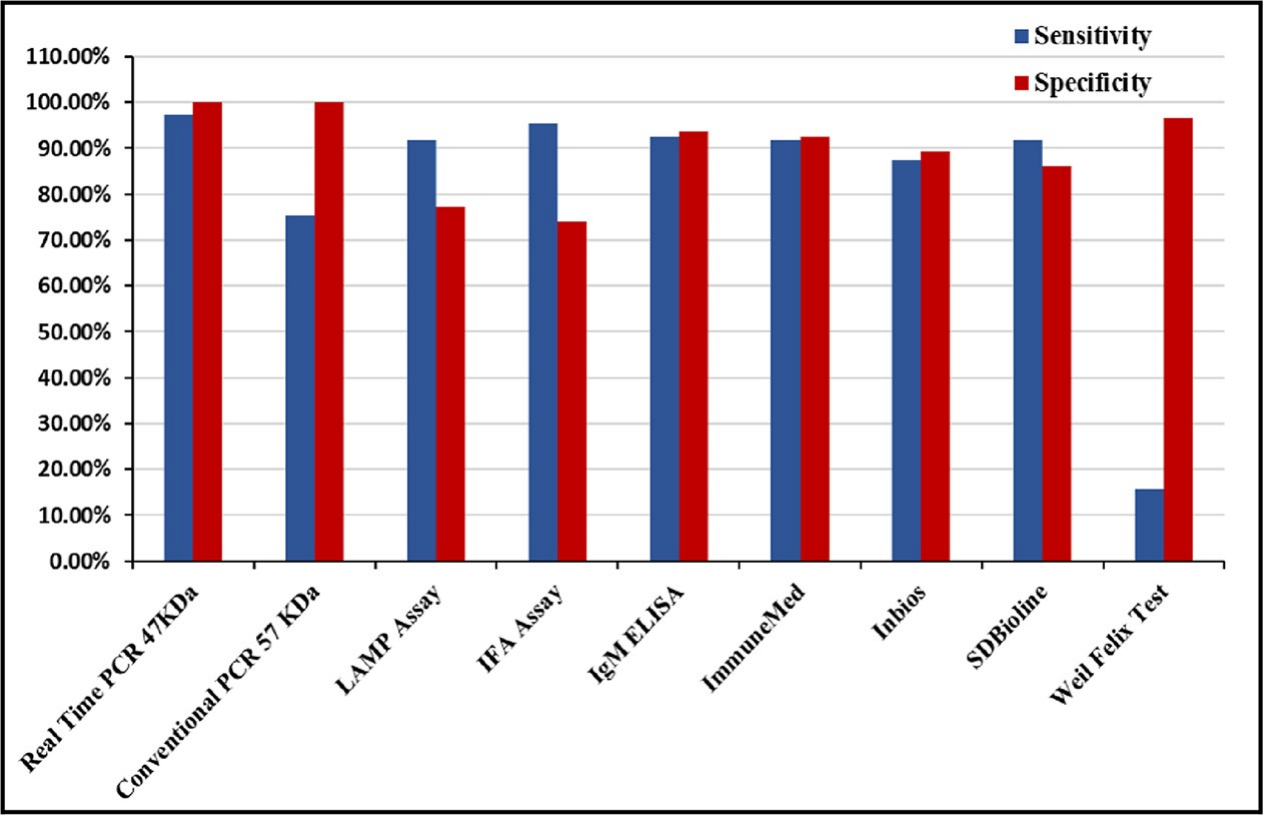
Graphical representation of the sensitivity and specificity of the different diagnostic test performed. Sensitivities and specificities of the molecular tests qPCR, cPCR and LAMP assay were found to be 97% & 100%, 75% & 100% and 92% & 77% respectively. The serological tests IFA assay, IgM ELISA and Weil Felix test have sensitivities and specificities of 95.6% & 74%, 94.2% & 93.67% and 15.82% & 96.6% respectively. Among the immunochromatographic RDTs Immune med, InBios and SD Bioline had sensitivities and specificities of 91.8% & 92.4%, 87.3% & 89.2% and 91.8 & 86.1% respectively.

The performances of the various diagnostic tests are depicted graphically in Fig 2 and their details are provided in Table 3. ELISA and RDT detecting IgM specific to *Orientia tsutsugamushi* had sensitivities and specificities of 92%, 94% and 92%, 92% respectively. Both these tests also demonstrated high positive likelihood ratios of 14.6 [OR:180·1; 75·45–429·7] and 12·08 [OR:135·7, CI: 59·91–307·37] respectively. IgM serology had a false positivity rate of 8% with other acute febrile illnesses such as dengue, leptospirosis and spotted fever. The Immunofluorescence test which is considered the serological gold standard had a noteworthy sensitivity of 96% but revealed modest specificity (74%). The Weil Felix test, the older conventional test utilized for assisting in the diagnosis of scrub typhus had very low sensitivity (16%) although it had good specificity (97%). However, Weil Felix test could not be performed on 11 controls due to inadequate sample volume. Among the healthy controls, LAMP assay and IFA showed a false positivity rate of 15% and 30% respectively. Among the immunochromatographic rapid diagnostic tests, SD Bioline had the highest false positivity rate of 12.5% followed by IgM ELISA, Immunemed RDT and InBios RDT of 5% each.

**Table 3:** Comparison of various diagnostic tests for scrub typhus.

Diagnostic Test (N-316; Cases-158,Controls-158)		Sensitivity (95%CI)	Specificity (95%CI)	PPV (95%CI)	NPV (95%CI)	Accuracy	Likelihood Ratio (+VE)	Likelihood Ratio (-VE)
**Blood PCR**	**Conventional PCR (56 KDa)**	75·32% (67.8–81.8)	100% (95.4–100)	100% (96.5–100)	80·20% (73.9–85.5)	87·66% (83.5–91.1)	-	0·25 (0.19–0.32)
	**Real time PCR (47 KDa)**	97·47% (93.8–99.3)	100% (96.5–100)	100% (96.5–100)	97·53% (93.6–99.3)	98·73% (96.8–99.7)	-	0·03 (0.01–0.07)
**LAMP Assay**		91·77% (86.3–95.5)	77·22% (69.9–83.5)	80·11% (73.5–85.7)	90·37% (84.1–94.8)	84·49% (80.02–88.3)	4·03 (3.09–5.39)	0·11 (0.06–0.18)
**IFA Assay**		95·57% (91.1–98.2)	74·05% (66.5–80.7)	78·65% (72.2–84.2)	94·35% (88.7–97.7)	84·81% (80.4–88.6)	3·68 (2.82–4.8)	0·06 (0.03–0.12)
**IgM ELISA**		92·41% (86.8–96)	93·67% (88.7–96.9)	93·59% (88.5–96.9)	92·50% (87.3–96.1)	93·04% (89.7–95.6)	14·6 (8–26.6)	0·08 (0.05–0.14)
**RDTs**	**SD Bioline**	91·77% (86.3–95.5)	86·08% (79.7–91.9)	86·83% (80.7–91.6)	91·28% (85.5–95.3)	88·92% (84.9–92.2)	6·59 (4.46–9.74)	0·10 (0.06–0.16)
	**InBios**	87·34% (81.1–92.1)	89·24% (83.3–93.6)	89·03% (83–93.5)	87·58% (81.5–92.2)	88·29% (84.2–91.6)	8·12 (5.16–12.77)	0·14 (0.09–0.21)
	**ImmuneMed**	91·77% (86.4–95.6)	92·41% (87–96)	92·36% (87.1–96)	91·82% (86.3–95.5)	92·09% (88.5–94.8)	12·08 (7–20.9)	0·09 (0.05–0.15)
*Weil Felix Text		15·82% (10.6–22.6)	96·60% (91.8–98.7)	83·33% (64.5–93.6)	51·64% (45.5–57.6)	54·75% (48.98–60.4)	4·65 (1.83–11.83)	0·87 (0.81–0.94)

N, sample size; 95%CI, 95% confidence interval; PPV, positive predictive value; NPV, negative predictive value. All the assays were performed on 158 cases and 158 controls except for Weil Felix test for which 158 cases and 147 controls were included.

Among the molecular tests, the best results were seen with the blood (buffy coat) qPCR which demonstrated excellent sensitivity (97%) and perfect specificity. Conventional PCR also demonstrated a perfect specificity but had a lower sensitivity (75%). The LAMP assay demonstrated a sensitivity of 92% with a specificity of 77%.

Twelve additional patients (7·6%) could be diagnosed with scrub typhus using PCR compared to IgM ELISA. Among these 12 patients, 8 (66·67%) had a duration of illness which is 7 days or less. Among the 158 scrub typhus cases, qPCR identified 154 (97·4%) whereas IgM ELISA detected 146 (92·4%).

Of the 4 patients missed by the qPCR, 3 of them had illness for 10 days or longer and the remaining one had duration of illness of 7 days. Among the 69 patients who had duration of illness less than or equal to 7 days, 61 tested positive for IgM ELISA (88·4%) and 68 were positive by qPCR (98·5%). Of the 146 patients positive for scrub typhus by IgM ELISA, 6 tested negative by ImmuneMed Scrub typhus Rapid RDT.

## Discussion

Scrub typhus, the most common rickettsial infection worldwide is often misdiagnosed due to its non-specific symptoms and signs. This study provides a comprehensive and deeper insight into the performance of various diagnostic tests for scrub typhus. ELISA and RDT detecting *O*.*tsutsugamushi- specifc* IgM were both noted to have tremendous discriminative potential with sensitivities and specificities of 92%, 94% and 92%, 92% respectively. PCR, particularly the qPCR using the 47kDa gene was identified to show the best results with a perfect specificity (100%) and a sensitivity of 97·5%.

In our study we used OD cut offs of 0·8 for IgM as positive for ELISA based on the results from the same endemic region [21]. Blacksell et al. in 2018 in Bangladesh determined the optimal optical density (OD) cut-off values for the diagnosis of scrub typhus using InBios ST IgM ELISA and concluded that the most appropriate cut-off OD to be within the range of 0·75–1·25 [22]. The use of the ST InBios IgM ELISA in Thailand to determine an admission diagnosis of scrub typhus using an ELISA cutoff at 0·5 OD (correlating to an IFA reciprocal titer cutoff of ≥1,600) revealed a sensitivity of 93% and a specificity of 91% [23]. A false positivity rate of 8% was noted when using IgM ELISA for scrub typhus. The IgM antibody has been known to show cross reactivity with many acute febrile illnesses, including leptospirosis, pulmonary tuberculosis, malaria and enteric fever [24, 25]. Non-specific binding of the IgM antibody, attributed to its pentavalent structure is probably the cause of this false positivity rate.

The IFA is considered as the serological gold standard in the diagnosis of the scrub typhus. In our study, the sensitivity and specificity of IFA were found to be 95% and 74% respectively. The specificity was much lower than that reported by Pote K et al. in their study done at Wardha (sensitivity and specificity of IgM IFA was 96·8% and 99·7% respectively) [26]. However the diagnostic confirmation in this study was less rigorous as a suboptimal gold standard of IFA was used adjusting the bias by latent class modeling to evaluate the performance of serological tests.

Among the three RDTs tested, the ImmuneMed Scrub typhus Rapid RDT showed the best results. This finding is similar to that of the study carried out in Korea which also revealed a superior sensitivity and specificity for ImmuneMed RDT (98·6%, 97·6%) compared to SD Bioline (84·4%, 96·3%) [27]. A meta-analysis performed by Saraswati K et al. published in 2018 suggested that the accuracy across data points of the same manufacturers varied across the studies [28]. The RDTs show great promise as a point of care test, particularly in the peripheral settings and emergency departments, where rapid diagnosis and prompt initiation of appropriate medication can prove lifesaving. They will be of practical benefit in primary and secondary care hospitals where tests such as ELISA for scrub typhus are not routinely carried out.

The Weil Felix test, the conventional agglutination test utilized in the diagnosis of rickettsial infections, is based on the cross reactive antibodies to different antigens of Proteus species. This attributes to poor sensitivity and specificity. Mahajan SK et al. in their study concluded that while Weil Felix agglutination test is not a very sensitive test, it is a particularly specific one [29]. In our study as well, we noted similar findings with the Weil Felix test demonstrating low sensitivities but high specificities at the cut off of ≥1:80. With better tests available, this test has now become obsolete.

Our study revealed that ELISA and RDTs were superior tests than IFA with greater specificity and near equal sensitivities. This is against the conventional thinking of the IFA being the serological gold standard. Despite being the standard reference serological test, IFA is cumbersome to do, requires expensive equipment and has inter-observer variations. It appears that the newer tests available are better suited for an accurate diagnosis of scrub typhus and may even replace IFA over time.

The pathognomic eschar was noted among 79% of cases which gives a good clinical clue to the diagnosis. This observation is higher than previously reported prevalence of 58–67% from this region [6, 30]. This is probably a result of increasing awareness among clinicians and a meticulous search for the eschar during physical examination. Eschars were not observed in the small number of spotted fever group rickettsiae (SFGR) among the controls.

In the initial 7 days of fever, serological tests may remain negative until the antibodies are formed. Patients presenting during this period would be missed if only serological tests are used to make the diagnosis. In this setting the value of PCR may be justified despite the expensive nature of the test. Our study revealed the best sensitivity and specificity with the qPCR and it was much superior to the cPCR. Kim et al. noted that the nested cPCR targeting 47KDa and 56KDa genes have similar sensitivities [16]. Therefore, the increased sensitivity noted with qPCR targeting 47kDa gene compared to the cPCR targeting 56kDa gene is more likely due to the higher sensitivity of the technique rather than the target. Furthermore, we collected the blood samples at admission prior to starting the antibiotics. The patients started on appropriate antibiotics, often becomes qPCR negative for blood in few days but remain positive for eschar and rash biopsies. In such situations, eschar / tissue samples for PCR will be beneficial. We also demonstrated that the sensitivity of the PCR can be increased by using the buffy coat rather than the whole blood. The necessity for a backup of standard reference laboratories to carry out the PCR as well as its cost is the key drawbacks to its routine use. The LAMP assay while being easier to perform and less expensive, revealed suboptimal results with a poor specificity and false positivity among the controls which can be attributed to the non specific binding of primers to high levels of host genomic DNA in the absence of specific targets [31]. Karthikeyan PA et al. based on their study carried at Puducherry, India concluded the opposite that the LAMP assay was highly specific (100%) and in addition had a good sensitivity of 89% in comparison to IgM ELISA, a suboptimal gold standard [32].

Antigen detection tests hold promise as the way forward in the early detection of scrub typhus. While such tests are currently unavailable, they will enable early detection of the disease and may prove to be more cost effective than PCR. More emphasis and further research in this area to develop such diagnostics will hopefully pave the way for more prompt and appropriate management of scrub typhus.

While our study had diverse acute febrile illnesses in the control group, the numbers of other rickettsial diseases included in the study were limited. The antibody cross reacting to other rickettsial infections needs additional evaluation, particularly in areas where other rickettsial diseases are highly prevalent.

## Conclusion

This study confirms that ELISA detecting *O*.*tsutsugamushi-specific* IgM has excellent sensitivity and specificity. The diagnostic accuracy of the IFA is suboptimal, challenging its role as the serological gold standard for the diagnosis of scrub typhus. The RDTs, particularly the ImmuneMed Scrub Typhus Rapid detecting the IgM antibodies, had very good sensitivity and specificity and may prove to be of great impact in the diagnosis of scrub typhus in primary and secondary health care settings. Given its perfect specificity and superior sensitivity, qPCR is preferred for diagnostic confirmation in reference laboratories and in particular for the diagnosis of early disease of less than 7 days duration. This study provides a comprehensive comparative evaluation of all currently available diagnostic tests for scrub typhus.

## References

[1] Watt G, Parola P. Scrub typhus and tropical rickettsioses. *Curr Opin Infect Dis*. 2003;16(5):429–36. 10.1097/00001432-200310000-00009 14501995

[2] Bonell A, Lubell Y, Newton PN, Crump JA, Paris DH. Estimating the burden of scrub typhus: A systematic review. *PLoSNegl Trop Dis*. 2017;11(9):e0005838 10.1371/journal.pntd.0005838 eCollection 2017 Sep. 28945755 PMC5634655

[3] Trowbridge P, P D, Premkumar PS, Varghese GM. Prevalence and risk factors for scrub typhus in South India. *Trop Med Int Health* 2017 2 7 10.1111/tmi.12853 28173608

[4] Ogawa M, Hagiwara T, Kishimoto T, et al Scrub typhus in Japan: Epidemiology and clinical features of cases reported in 1998. *Am J Trop Med Hyg* 2002 67:162–165. 10.4269/ajtmh.2002.67.162 12389941

[5] Kundavaram AP, Jonathan AJ, SD Nathaniel, GM Varghese. Eschar in Scrub typhus: A valuable clue to diagnosis. *J Postgrad Med* 2013 59:177–78. 10.4103/0022-3859.118033 24029193

[6] Varghese GM, Trowbridge P, Janardhanan J et al Clinical Profile and Improving Mortality Trend of Scrub Typhus in South India. *Int J Infect Dis*. 2014; 23: 39–43. 10.1016/j.ijid.2014.02.009 24661931

[7] Xu G, Walker DH, Jupiter D, Melby PC, Arcari CM. A review of the global epidemiology of scrub typhus. *PLoSNegl*. *Trop*. *Dis* 2017 11, e0006062 10.1371/journal.pntd.0006062 29099844 PMC5687757

[8] Peter JV, Sudarsan TI, Prakash JA, Varghese GM. Severe scrub typhus infection: Clinical features, diagnostic challenges and management. *World J Crit Care Med* 2015;4(3):244–250. 10.5492/wjccm.v4.i3.244 26261776 PMC4524821

[9] Jang WJ, Huh MS, Park KH, Choi MS, Kim IS. Evaluation of an Immunoglobulin M Capture Enzyme-Linked Immunosorbent Assay for Diagnosis of *Orientia tsutsugamushi* Infection. *ClinDiagn Lab Immunol*. 2003; 10 (3): 394–398. 10.1128/CDLI.10.3.394-398.2003 PMC154952 12738637

[10] Blacksell SD, Jenjaroen K, Phetsouvanh R et al Accuracy of AccessBio Immunoglobulin M and Total Antibody Rapid Immunochromatographic Assays for the Diagnosis of Acute Scrub Typhus Infection. *Clin Vaccine Immunol* 2010;17:263–266. 10.1128/CVI.00448-08 20016046 PMC2815529

[11] Blacksell SD, Jenjaroen K, Phetsouvanh R et al Accuracy of rapid IgM-based immunochromatographic and immunoblot assays for diagnosis of acute scrub typhus and murine typhus infections in Laos. *Am J Trop Med Hyg* 2010;83:365–369. 10.4269/ajtmh.2010.09-0534 20682883 PMC2911186

[12] Blacksell SD, Paris DH, Chierakul W et al Prospective evaluation of commercial antibody-based rapid tests in combination with a loop-mediated isothermal amplification PCR assay for detection of Orientia tsutsugamushi during the acute phase of scrub typhus infection. *Clin Vaccine Immunol* 2012;19:391–395 10.1128/CVI.05478-11 22219313 PMC3294598

[13] Koh GC, Maude RJ, Paris DH, Newton PN, Blacksell SD. Diagnosis of scrub typhus. *Am J Trop Med Hyg* 2010; 82: 368–70. 10.4269/ajtmh.2010.09-0233 20207857 PMC2829893

[14] Premaratna R, Blanton LS, Samaraweera DN et al Genotypic characterization of *Orientia tsutsugamushi* from patients in two geographical locations in Sri Lanka. *BMC Infect Dis* 2017;17:67 10.1186/s12879-016-2165-z 28086810 PMC5237229

[15] Tantibhedhyangkul W, Wongsawat E, Silpasakorn S et al Use of Multiplex Real-Time PCR To Diagnose Scrub Typhus. *J ClinMicrobiol*. 2017;55(5):1377–1387. 10.1128/JCM.02181-16 28202789 PMC5405255

[16] Kim DM, Park G, Kim HS et al Comparison of conventional, nested, and real-time quantitative PCR for diagnosis of scrub typhus. *J Clin Microbiol*. 2011;49(2):607–612. 10.1128/JCM.01216-09 21068287 PMC3043474

[17] Paris DH, Blacksell SD, Nawtaisong P, et al Diagnostic accuracy of a loop-mediated isothermal PCR assay for detection of Orientia tsutsugamushi during acute Scrub Typhus infection. *PLoS Negl Trop Dis*. 2011;5(9):e1307 10.1371/journal.pntd.0001307 21931873 PMC3172190

[18] Varghese GM, Janardhanan J, Mahajan SK, Tariang D, Trowbridge P, Prakash JAJ, David T, Sathendra S, Abraham OC. Molecular Epidemiology and Genetic Diversity of *Orientia tsutsugamushi* from Patients with Scrub Typhus in India. Emerg Infect Dis. 2015; 21: 64–69. 10.3201/eid2101.140580 25530231 PMC4285260

[19] Jiang J, Chan TC, Temenak JJ, Dasch GA, Ching WM, Richards AL. Development of a quantitative real-time polymerase chain reaction assay specific for Orientia tsutsugamushi. *Am J Trop Med Hyg* 2004;70(4):351–6. 15100446

[20] Mori Y, Nagamine K, Tomita N, Notomi T. Detection of loop-mediated isothermal amplification reaction by turbidity derived from magnesium pyrophosphate formation. *Biochem*.*Biophys*. *Res*. *Commun* 2001 289:150–154 10.1006/bbrc.2001.5921 11708792

[21] Varghese GM, Rajagopal VM, Trowbridge P, Purushothaman D, Martin SJ. Kinetics of IgM and IgG antibodies after scrub typhus infection and the clinical implications. *Int J Infect Dis*. 2018;71:53–55. 10.1016/j.ijid.2018.03.018 29653201 PMC5985369

[22] Blacksell S, Kingston H, Tanganuchitcharnchai A et al Diagnostic Accuracy of the InBios Scrub Typhus Detect ELISA for the Detection of IgM Antibodies in Chittagong, Bangladesh. *Trop Med Infect Dis* 2018;3(3):95 10.3390/tropicalmed3030095 30274491 PMC6160969

[23] Blacksell SD, Tanganuchitcharnchai A, Nawtaisong P, et al Diagnostic Accuracy of the InBios Scrub Typhus Detect Enzyme-Linked Immunoassay for the Detection of IgM Antibodies in Northern Thailand. *Clin Vaccine Immunol* 2015;23(2):148–154. Published 2015 Dec 9. 10.1128/CVI.00553-15 26656118 PMC4744921

[24] Gupta N, Chaudhry R, Mirdha B et al Scrub typhus and leptospirosis: The fallacy of diagnosing with IgM enzyme linked immunosorbant assay. *J Microb Biochem Technol* 2016;8:071–5

[25] Prakash JA, Abraham OC, Mathai E. Evaluation of tests for serological diagnosis of scrub typhus. *Trop Doct* 2006;36:212–213. 10.1258/004947506778604715 17034691

[26] Pote K, Narang R, Deshmukh P. Diagnostic performance of serological tests to detect antibodies against acute scrub typhus infection in central India. *Indian J Med Microbiol* 2018;36:108–12. 10.4103/ijmm.IJMM_17_405 29735837

[27] Kim YJ, Park S, Premaratna R et al Clinical Evaluation of Rapid Diagnostic Test Kit for Scrub Typhus with Improved Performance. *J Korean Med Sci* 2016;31(8):1190–1196. 10.3346/jkms.2016.31.8.1190 27478327 PMC4951546

[28] Saraswati K, Day NPJ, Mukaka M, Blacksell SD. Scrub typhus point-of-care testing: A systematic review and meta-analysis. *PLoS Negl Trop Dis* 2018;12(3):e0006330 10.1371/journal.pntd.0006330 29579046 PMC5892940

[29] Mahajan S, Kashyap R, Kanga A, Sharma V, Prasher BS, Pal LS. Relevance of Weil-Felix test in diagnosis of scrub typhus in India. *J Assoc Physicians India* 2006;54:619–621. 16941792

[30] Ganesh R, Suresh N, Pratyusha LL, Janakiraman L, Manickam M, Andal A. Clinical profile and outcome of children with scrub typhus from Chennai, South India. *Eur J Pediatr*. 2018;177(6):887–890. 10.1007/s00431-018-3143-9 29637374

[31] Dittrich S, Castonguay-Vanier J, Moore CE, Thongyoo N, Newton PN, Paris DH. Loop-mediated isothermal amplification for Rickettsia typhi (the causal agent of murine typhus): problems with diagnosis at the limit of detection. *J Clin Microbiol*. 2014;52 (3):832–838. 10.1128/JCM.02786-13 24371248 PMC3957756

[32] Karthikeyan PA, Hoti SL, Kanungo R. Evaluation of loop-mediated isothermal amplification assay for detection of scrub typhus in patients with acute febrile illness presenting to a Tertiary Care Center in Puducherry, India. *J Lab Physicians* 2019;11(1):82–86. 10.4103/JLP.JLP_148_18 30983808 PMC6437831

